# Diffusion-weighted MR imaging histogram analysis in HIV positive and negative patients with primary central nervous system lymphoma as a predictor of outcome and tumor proliferation

**DOI:** 10.18632/oncotarget.27800

**Published:** 2020-11-10

**Authors:** Bilal Khan, Insun Chong, Quinn Ostrom, Sara Ahmed, Dima Dandachi, Aikaterini Kotrotsou, Rivka Colen, Fanny Morón

**Affiliations:** ^1^Department of Radiology, Baylor College of Medicine, Houston, TX, USA; ^2^Department of Medicine, Section of Epidemiology and Population Sciences, Dan L. Duncan Comprehensive Cancer Center, Baylor College of Medicine, Houston, TX, USA; ^3^Department of Cancer Systems Imaging, Division of Diagnostic Imaging, The University of Texas at MD Anderson Cancer Center, Houston, TX, USA; ^4^Department of Diagnostic Radiology, Division of Diagnostic Imaging, The University of Texas at MD Anderson Cancer Center, Houston, TX, USA; ^5^Department of Medicine, Division of Infectious Diseases, University of Missouri, Columbia, MO, USA; ^6^Department of Radiology, Neuroradiology Division, University of Pittsburgh School of Medicine, Pittsburgh, PA, USA

**Keywords:** primary CNS lymphoma, lymphoma, MRI, diffusion weighted, HIV

## Abstract

Introduction: Ki-67 expression, a marker of tumor proliferation, is considered a prognostic factor in primary CNS lymphoma (PCNSL). Apparent diffusion coefficient (ADC) parameters have also been proposed as imaging biomarkers for tumor progression and proliferative activity in various malignancies. The aim of this study is to investigate the correlation between ADC parameters, Ki-67 expression, overall survival (OS) and progression free survival (PFS) in PCNSL.

Materials and Methods: Patients diagnosed with PCNSL at MD Anderson Cancer Center between Mar 2000 and Jul 2016 and at Ben Taub Hospital between Jan 2012 and Dec 2016 were retrospectively studied. Co-registered ADC maps and post-contrast images underwent whole tumor segmentation. Normalized ADC parameters (nADC) were calculated as the ratio to normal white matter. Percentiles of nADC were calculated and were correlated with Ki-67 using Pearson’s correlation coefficient and clinical outcomes (OS and PFS) using Cox proportional hazards models.

Results: Selection criteria yielded 90 patients, 23 patients living with HIV (PLWH) and 67 immunocompetent patients. Above median values for nADC_mean_, nADC_15_, nADC_75_ and nADC_95_ were associated with improved OS in all patients (*p* < 0.05). Above median values for nADC_min_, nADC_mean_, nADC_1_, nADC_5_ and kurtosis were associated with improved PFS in all patients (*p* < 0.05). In patients with available Ki-67 expression data (*n* = 22), nADC_mean_, nADC_15_ and nADC_75_ inversely correlated with Ki-67 expression (*p* < 0.05). For PLWH, there was no correlation between ADC parameters and Ki-67 expression or clinical outcomes.

Conclusions: ADC histogram analysis can predict tumor proliferation and survival in immunocompetent patients with PCNSL, but with limited utility in PLWH.

## INTRODUCTION

Primary central nervous system lymphoma (PCNSL) is a rare subgroup of non-Hodgkin lymphoma confined to the central nervous system, with more than 90% of cases classified as Diffuse Large B-cell Lymphoma [1, 2]. Immunosuppressed states have been an established risk factor for PCNSL, with HIV representing a leading factor for the rise in the incidence of PCNSL [2]. Patients living with HIV (PLWH) represent an important population to include in investigations related to PCNSL, as the association with the Epstein Barr virus highlights PCNSL in PLWH is a distinct entity from sporadic cases [3, 4]. While emerging cyto-reductive treatment regimens demonstrate promising results, PCNSL recurrence rate remains high due to the restricted penetration of treatment agents through the protective blood-brain barrier [5–7]. As efficacious therapy strategies continue to evolve and clinical outcomes remain poor, the need to identify prognostic biomarkers for potential risk-stratified and optimized treatment in PCNSL remains an area of further exploration.

Magnetic resonance imaging (MRI) is the modality of choice for imaging CNS lymphomas [8]. Conventional gadolinium enhanced sequences can provide details of the tumor location, infiltration, enhancement pattern and mass effect but without adequately reflecting tumor histopathology [9]. However, diffusion weighted imaging (DWI) and corresponding apparent diffusion coefficient (ADC) maps can provide a representation of the cellular microenvironment [10, 11] with several studies demonstrating that ADC values can predict tumor cellularity across various neoplasms, including lymphomas [10–14]. Expression of tumor Ki-67, a cellular biomarker for proliferative activity, has been shown correlate with overall survival (OS) and progression-free survival (PFS) in PCNSL patients [15–17]. Studies have also suggested that ADC values from various malignancies correlate with the expression of Ki-67 [13, 14, 18–26]. Therefore, ADC values may serve as a potential noninvasive method to prognosticate and monitor response to treatment in PCNSL. These studies, however, were limited due to the small sample sizes (< 30) or neglected to investigate the effect of immune status, as PLWH represent a well-established, clinically distinct, subset of patients with PCNSL. Prior studies also did not incorporate whole tumor volume segmentation with T1 contrast co-registration, which is the most reliable and reproducible method of ADC histogram acquisition [27]. In a recent similar study of whole tumor histogram analysis in PCNSL performed by the authors of this study [28], multiple ADC parameters were inversely correlated with Ki-67 expression and associated with poorer OS. However, tumor segmentation was performed using only the ADC sequence with the potential inclusion of intra-tumoral necrosis, hemorrhage or regions outside of the actual solid tumor that would otherwise have been excluded with contrast co-registration, ultimately providing a suboptimal representation of true tumor parenchyma.

The primary aim of this study is to more comprehensively evaluate the relationship between ADC calculations with tumor Ki-67 expression and clinical outcomes (OS and PFS) using a larger patient sample with the inclusion of PLWH and whole tumor segmentation with T1 post contrast co-registration. Our hypothesis is that ADC values will inversely correlate with Ki-67 expression and that tumors with higher ADC values above the median will have improved OS and PFS.

## RESULTS

### Patient demographics

90 patients met the inclusion criteria. Of these, 32 were from BTH (36%) and 58 were from MDACC (64%). 23 patients were HIV positive (26%) while 67 patients were HIV negative (74%). Of the 23 patients who were HIV positive, only 1 patient (4.3%) was from MDACC (*p* < 0.001). Further demographic information is summarized in Supplementary Table 1. One patient was excluded in the Cox Proportional Hazards Models calculations due to lack of available ECOG score.

### Qualitative imaging characteristics

Ring enhancement was more common in PLWH than immunocompetent patients (*p* < 0.001). PLWH were more likely to have multiple tumors while immunocompetent patients were more likely to have single lesions (*p* = 0.01). Tumor hemorrhage was also more common in PLWH, but not significant (*p* = 0.14). Further qualitative imaging characteristics are summarized in Supplementary Table 1.

### Correlations between ADC parameters and Ki-67

Ki-67 expression data was available for a subset of patients (22, 24.4% of total). There were no significant correlations identified between Ki-67 expression and any ADC histogram parameter (Table 1) in all patients. After lesions from PLWH were excluded (*n* = 19), statistically significant inverse correlations were observed with nADC_mean_ (*r* = –0.49, *p* = 0.03), nADC_15_ (*r* = –0.58, *p* = 0.009), and nADC_75_ (*r* = –0.48, *p* = 0.04). There were no significant correlations in the HIV positive subset.

**Table 1:** Correlation with Ki-67 in all patients and patients with and without HIV

Parameter	All patients (*n* = 22)		HIV positive (*n* = 3)		HIV negative (*n* = 19)	
	*r*	*p*-value	*r*	*p*-value	*r*	*p*-value
Skewness	−0.130	0.5641	−0.666	0.5361	−0.102	0.6785
Kurtosis	−0.226	0.3125	−0.901	0.2859	−0.167	0.4957
nADC_Min_	0.244	0.2737	0.973	0.1486	0.193	0.4284
nADC_Max_	−0.259	0.2442	0.725	0.4834	−0.323	0.1775
nADC_Mean_	−0.237	0.2876	0.955	0.1928	−0.494	0.0317^*^
nADC_1_	0.290	0.1907	0.702	0.5050	0.241	0.3204
nADC_5_	0.199	0.3744	0.617	0.5766	0.130	0.5946
nADC_15_	−0.394	0.0695	0.828	0.3787	−0.580	0.0092^*^
nADC_75_	−0.349	0.1118	0.942	0.2185	−0.477	0.0391^*^
nADC_95_	−0.267	0.2297	0.991	0.0849	−0.358	0.1325
nADC_99_	−0.148	0.5122	0.921	0.2551	−0.175	0.4743

Results considered significant (^*^) when *p* < 0.05.

### ADC parameters and overall survival

**Figure 1:**
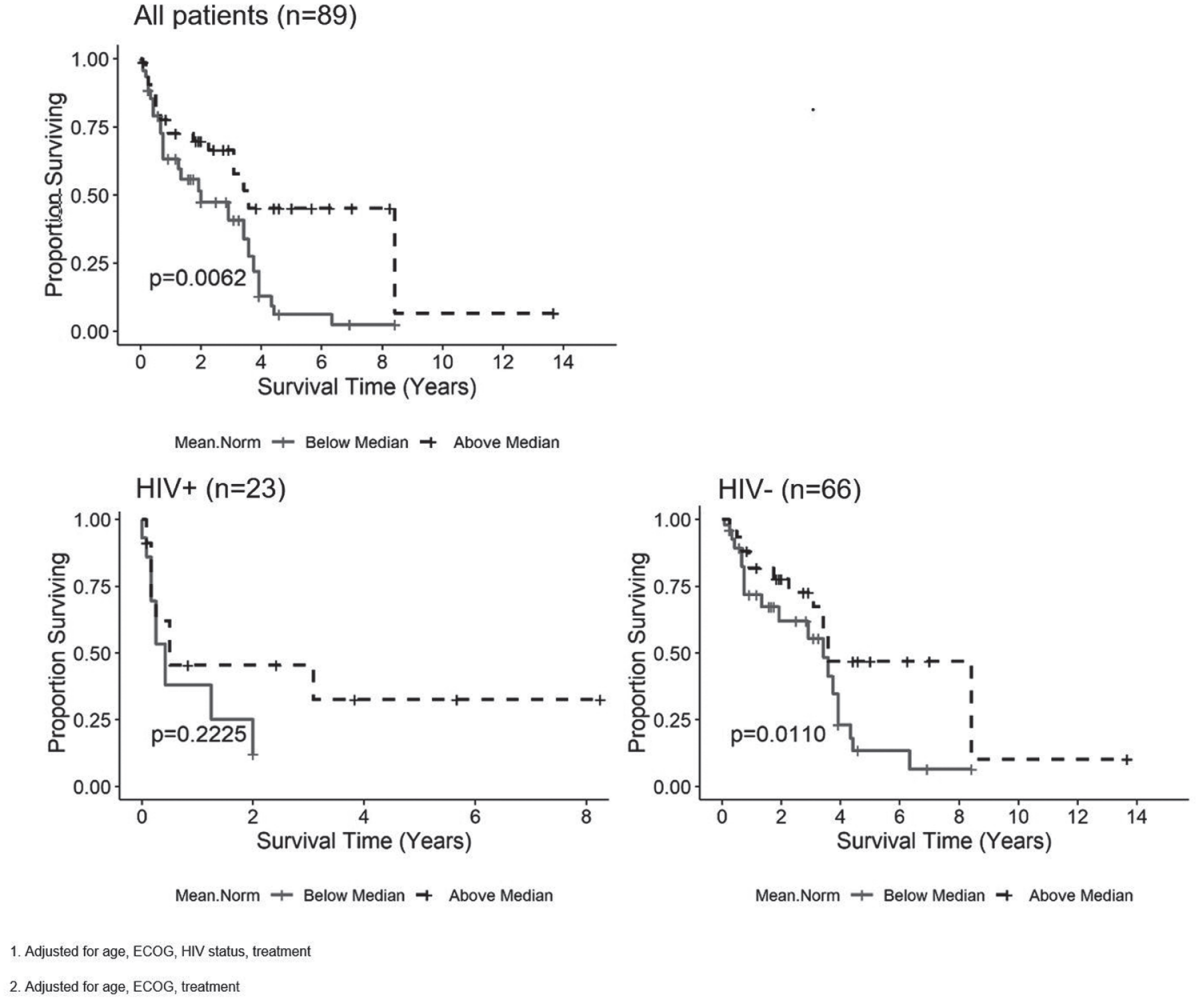
Kaplan-Meier curves for the nADC_mean_ parameter and overall survival (OS) in all patients^1^, patients living with HIV^2^ (PLWH) and HIV negative patients^2^. Tumors with ADC values above the median for nADC_mean_ are associated with improved OS in all patients and HIV negative patients, but not in PLWH.

Four ADC histogram parameters were found to have a statistically significant relationship with OS in all patients after adjustment for age, HIV status, ECOG, and treatment (Table 2). Values above the median for nADC_mean_ (HR = 0.47, 95% CI = 0.253–0.855, *p* = 0.006), nADC_15_ (HR = 0.879, 95% CI = 0.456–1.695, *p* = 0.04), nADC_75_ (HR = 0.581, 95% CI = 0.294–1.148, *p* = 0.02) and nADC_95_ (HR = 0.788, 95% CI = 0.427–1.453, *p* = 0.04) were associated with improved OS. When analysis was stratified based on HIV status, values above the median for nADC_mean_ (HR = 0.461, 95% CI = 0.21–1.012, *p* = 0.01), nADC_75_ (HR = 0.674, 95% CI = 0.286–1.585, *p* = 0.02) and nADC_95_ (HR = 0.799, 95% CI = 0.368–1.737, *p* = 0.06) were significant predictors for improved OS in HIV negative patients, while there were no significant associations with PLWH. Figure 1 illustrates the OS curve for nADC_mean_.

**Table 2:** Hazard ratios for overall survival in all patients and patients with and without HIV

Feature	All patients (*n* = 89)^1^		HIV+ (*N* = 23)^2^		HIV- (*n* = 66)^2^	
	*p*-value	HR (95% CI)	*p*-value	HR (95% CI)	*p*-value	HR (95% CI)
Skewness	0.6817	1.463 (0.785–2.728)	0.6137	2.022 (0.661–6.185)	0.2522	1.555 (0.688–3.517)
Kurtosis	0.2677	1.427 (0.708–2.877)	0.2478	2.429 (0.494–11.956)	0.4393	1.383 (0.598–3.199)
nADC_Min_	0.8748	0.789 (0.448–1.391)	0.7748	0.909 (0.302–2.738)	0.2999	0.692 (0.346–1.383)
nADC_Max_	0.0880	0.737 (0.405–1.342)	0.3526	0.574 (0.17–1.943)	0.6768	0.874 (0.428–1.782)
nADC_Mean_	0.0062^*^	0.465 (0.253–0.855)	0.2225	0.481 (0.162–1.427)	0.0110^*^	0.461 (0.21–1.012)
nADC_1_	0.2265	0.721 (0.409–1.268)	0.6766	0.698 (0.227–2.145)	0.2771	0.656 (0.324–1.328)
nADC_5_	0.2549	0.860 (0.49–1.511)	0.6904	1.062 (0.381–2.959)	0.3075	0.709 (0.354–1.422)
nADC_15_	0.0364^*^	0.879 (0.456–1.695)	0.4947	1.068 (0.307–3.718)	0.0691	0.908 (0.377–2.188)
nADC_75_	0.0149^*^	0.581 (0.294–1.148)	0.6153	0.463 (0.131–1.634)	0.0156^*^	0.674 (0.286–1.585)
nADC_95_	0.0358^*^	0.788 (0.427–1.453)	0.6594	1.051 (0.291–3.799)	0.0567	0.799 (0.368–1.737)
nADC_99_	0.4637	0.562 (0.289–1.092)	0.2643	0.412 (0.084–2.026)	0.2898	0.482 (0.213–1.090)

Results considered significant (^*^) when *p* < 0.05. ^1^Adjusted for age, ECOG, HIV status, treatment, ^2^Adjusted for age, ECOG, treatment.

### ADC parameters and progression-free survival

**Figure 2:**
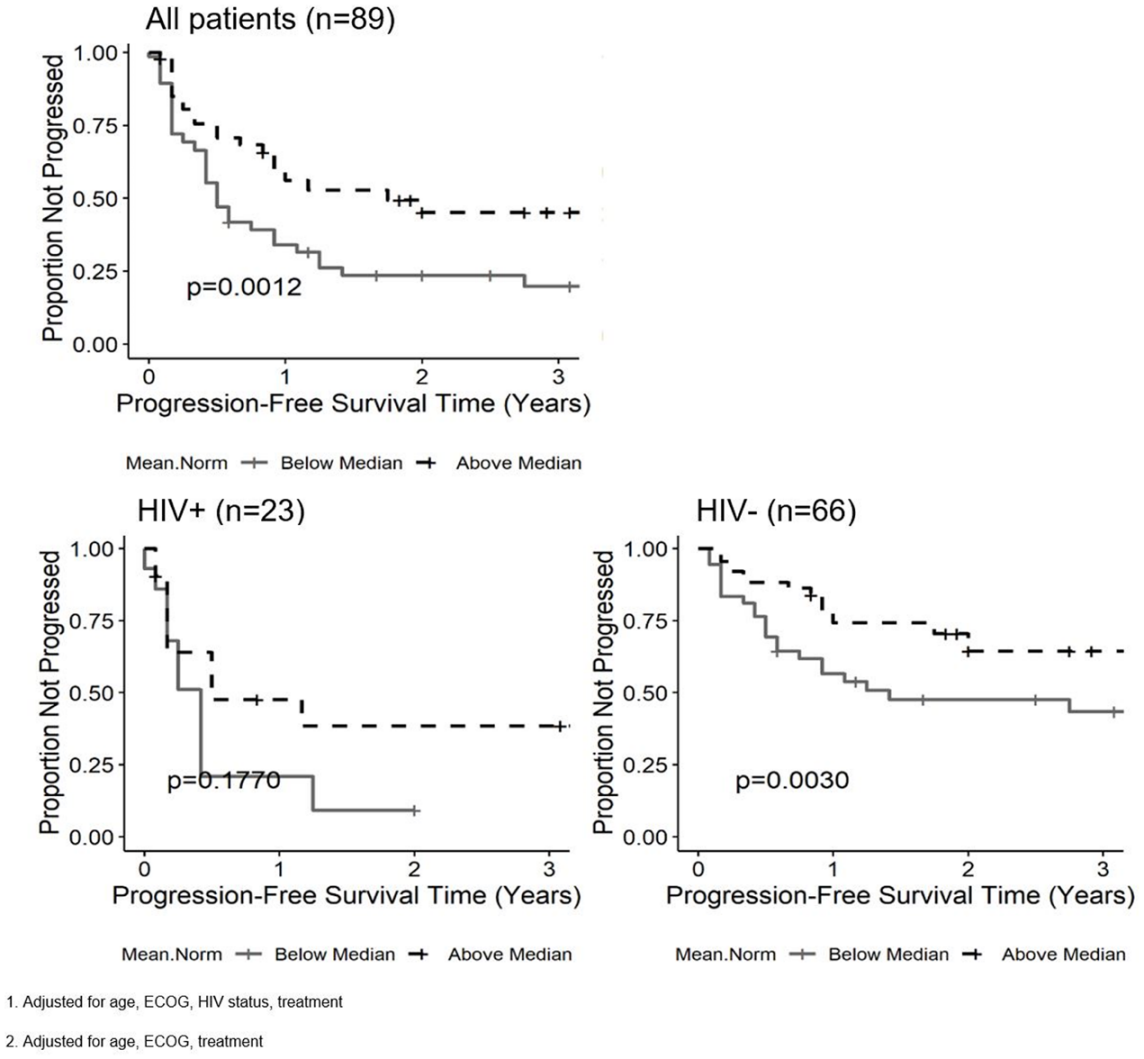
Kaplan-Meier curves for the nADC_mean_ parameter and progression free survival (PFS) in all patients^1^, patients living with HIV^2^ (PLWH) and HIV negative patients^2^. Tumors with ADC values above the median for nADC_mean_ are associated with improved PFS in all patients and HIV negative patients, but not in PLWH.

Five ADC histogram parameters were found to have a statistically significant relationship with PFS in all patients after adjustment for age, HIV status, ECOG and treatment (Table 3). Values above the median for nADC_min_ (HR = 0.557, 95% CI = 0.316–0.983, *p* = 0.03), nADC_mean_ (HR = 0.462, 95% CI = 0.255–0.838, *p* = 0.001), nADC_1_ (HR = 0.436, 95% CI = 0.244–0.780, *p* = 0.004), nADC_5_ (HR = 0.484, 95% CI = 0.272–0.862, p = 0.006) and ADC histogram kurtosis (HR = 2.35, 95% CI = 1.204–4.588, *p* = 0.008) were significant predictors for improved PFS. Values above the median for nADC_75_ were marginally significant (HR = 0.618, 95% CI = 0.326–1.170, *p* = 0.06). When analysis was limited to HIV negative patients, values above the median of five ADC parameters were significant predictors for improved PFS, including nADC_min_ (HR = 0.427, 95% CI = 0.21–0.869, *p* = 0.005), nADC_mean_ (HR = 0.437, 95% CI = 0.206–0.927, *p* = 0.003), nADC_1_ (HR = 0.35, 95% CI = 0.171–0.718, *p* = 0.002), nADC_5_ (HR = 0.393, 95% CI = 0.195–0.794, *p* = 0.003) and nADC_75_ (HR = 0.583, 95% CI = 0.268–1.27, *p* = 0.03). In the HIV positive subset, there were no significant correlations with any ADC parameter and PFS. Figure 2 illustrates the PFS curve for nADC_mean_.

**Table 3:** Hazard ratios for progression free survival in all patients and patients with and without HIV

Feature	All patients (*n* = 89)^1^		HIV+ (*N* = 23)^2^		HIV- (*n* = 66)^2^	
	*p*-value	HR (95% CI)	*p*-value	>HR (95% CI)	*p*-value	HR (95% CI)
Skewness	0.1627	2.026 (1.131–3.627)	0.9491	2.839 (0.851–9.47)	0.1055	1.6 (0.778–3.29)
Kurtosis	0.0082^*^	2.35 (1.204–4.588)	0.1869	4.313 (0.537–34.662)	0.0759	1.848 (0.879–3.883)
nADC_Min_	0.0313^*^	0.557 (0.316–0.983)	0.9330	0.835 (0.27–2.585)	0.0053^*^	0.427 (0.21–0.869)
nADC_Max_	0.4497	0.799 (0.461–1.385)	0.5308	0.8 (0.254–2.522)	0.6771	0.803 (0.409–1.576)
nADC_Mean_	0.0012^*^	0.462 (0.255–0.838)	0.1770	0.448 (0.151–1.325)	0.0030^*^	0.437 (0.206–0.927)
nADC_1_	0.0043^*^	0.436 (0.244–0.78)	0.4448	0.636 (0.2–2.023)	0.0018^*^	0.35 (0.171–0.718)
nADC_5_	0.0063^*^	0.484 (0.272–0.862)	0.4351	0.845 (0.3–2.383)	0.0033^*^	0.393 (0.195–0.794)
nADC_15_	0.2584	1.183 (0.625–2.238)	0.6675	1.533 (0.39–6.022)	0.1990	0.947 (0.441–2.035)
nADC_75_	0.0569	0.618 (0.326–1.17)	0.7717	0.482 (0.135–1.719)	0.0248^*^	0.583 (0.268–1.27)
nADC_95_	0.1482	0.889 (0.504–1.568)	0.9131	1.416 (0.407–4.931)	0.0864	0.714 (0.35–1.455)
nADC_99_	0.3223	0.353 (0.188–0.664)	0.2295	0.232 (0.029–1.863)	0.4752	0.398 (0.197–0.803)

Results considered significant (^*^) when *p* < 0.05. ^1^Adjusted for age, ECOG, HIV status, treatment. ^2^Adjusted for age, ECOG, treatment.

### Additional factors impacting overall survival and progression-free survival

HIV status was associated with poorer OS (median survival of 6 months versus 43 months, *p* = 0.003). ECOG score of 0–1 was associated with improved OS for all patients (median survival of 47 months versus 8 months, *p* < 0.001). Individuals with lesion hemorrhage had poorer OS (median survival of 21 months versus 30 months, *p* = 0.01) and PFS (median time to recurrence 22 months versus 30 months, *p* = 0.05). These findings are summarized on Supplementary Table 1.

## DISCUSSION

Our results suggest that multiple normalized ADC parameters inversely correlate with tumor Ki-67 expression in HIV negative patients, specifically nADC_mean_, nADC_15_ and nADC_75_. This inverse relationship between ADC parameters and Ki-67 expression concurs with existing studies [19, 20, 22–26, 29, 30]. Schob and colleagues (2016) examined 21 patients with PCNSL using single ROI for ADC quantification and found inverse correlations between Ki-67 expression and multiple ADC parameters [19]. While our correlations were only significant in the HIV negative subset, the findings by Schob and colleagues also only included immunocompetent patients. Further work by Schob et al. (2018) found that only the ninetieth percentile (ADC_90_) of whole tumor diffusion profiling showed an inverse correlation with the expression of Ki-67 [20]. Our findings suggest that additional ADC parameters not previously identified may reflect tumor proliferation, which may indicate a higher sensitivity of ADC analysis to predict proliferation than previously thought. A potential explanation may be related to both our larger patient sample size and the use of whole tumor segmentation with post-contrast co-registration, which has been shown to be a more reliable and reproducible method of segmentation compared to the singular ROI method utilized by prior studies [27].

Our study suggests that ADC values above the median of multiple normalized ADC parameters were significant predictors of improved OS and PFS in immunocompetent patients with PCNSL. We postulate that tumors with higher ADC values reflect less cellularity, as the inverse relationship between tumor cellularity and ADC values has been shown in a broad spectrum of malignancies, including PCNSL [10, 11, 18] and may imply less aggressive tumors that are more responsive to existing treatment. Existing studies in the literature concur with our findings in immunocompetent patients with PCNSL. Barajas et al. (2010) found that the low ADC group (defined by ADC_25_) was associated with shorter PFS and OS and the high ADC group was found to have improved OS and PFS [18]. Valles et al. (2013) also demonstrated that lesions with ADC values in the low ADC parameter were associated with increased progression and death compared to the high ADC group [21]. Our findings not only substantiate the literature supporting the use of ADC as an imaging biomarker for prognosis, but also suggest that additional ADC parameters not previously identified as significant may also predict outcomes. Furthermore, prediction of survival outcomes based on ADC values remained significant even when adjusting for treatment. This suggests that the pre-treatment MR exam with DWI and T1 post-contrast sequences should be sufficient to predict OS and PFS in immunocompetent PCNSL patients.

The current study investigates the impact of immune status on ADC histogram analysis for tumor proliferation expression and patient outcomes, an area largely unexplored in the current literature. In this study, ADC values from PLWH did not show any correlation with Ki-67 expression or with patient outcomes. A possible explanation may be that PCNSL in immunosuppressed patients more often exhibit necrotic regions and may develop spontaneous hemorrhage compared to HIV negative patients [8, 31]. We hypothesize that the tumoral necrosis characteristic of PCNSL in PLWH results in decreased tumor cellularity and viable tumor, thereby altering the tumoral ADC profile and ultimately the relationship of ADC to clinical outcomes and histology. Hemorrhage is also known to alter the tumor micro-environment, leading to changes in ADC values based on the age of the hemorrhage [32–34]. Our similar recently published work demonstrated significant associations between multiple ADC parameters and poorer OS, in addition to significant inverse correlations with Ki-67 expression, even in PLWH [28]. However, a major limitation was the lack of co-registration of the ADC and T1 post-contrast sequences during segmentation. Segmentation of the ADC sequence alone in that study may have included areas of intra-tumoral necrosis, hemorrhage, or regions outside of the enhancing tumor margin, resulting in variable and inaccurate tumor ADC histogram characterization. In the current study using co-registration, while areas of necrosis and hemorrhage were excluded during segmentation based on imaging features, microscopic necrosis or hemorrhage beyond the resolution of imaging may have been included. Additionally, the lack of correlation between ADC parameters and Ki-67 expression in PLWH may be the result of the small sample size of PLWH with available Ki-67 data (*n* = 3). The very small sample size results in a significant limitation in drawing a correlation between ADC parameters and tumor proliferative activity in PLWH, necessitating further evaluation with larger sample sizes in potential future studies. Overall, ADC histogram profiling may have a limited role in patients with immunosuppression, although the imaging, pathological and clinical correlation of PCNSL in PLWH is still a developing area of research.

This study identified additional qualitative imaging features and clinical factors to further expand the knowledge of PCNSL outside of ADC histogram analysis. Lesions from PLWH were more likely to be ring-enhancing and multi-centric compared to lesions in the immunocompetent, as has been well established [8, 35]. HIV status was associated with poorer overall survival, which also concurs with prior findings [36]. Our results substantiate the existing knowledge that the imaging and clinical features for PCNSL in PLWH and immunocompetent patients are distinct entities [3, 4, 8]. Lesion hemorrhage, higher ECOG scores and the absence of treatment with autologous stem cell transplantation were also associated with poorer outcomes when adjusting for HIV status, similar to other established studies [36–38]. This confirms additional prognostic factors to consider in the treatment of PCNSL in conjunction with ADC histogram analysis.

There are various limitations to our study. Although our patient population appears representative of a large group, our sample size was relatively small, though remains larger than similar radiological publications. The small sample size of available Ki-67 data is a potential cause for lack of statistical significance for correlations between the various ADC parameters when adjusting for the false discovery rate, although there still appears to be a trend between ADC parameters and Ki-67 that warrants further investigation. Secondly, the retrospective and multi-center nature of the study and the use of multiple vendors and magnet strengths resulted in non-uniform image acquisition parameters, which alters ADC values [39]. This, however, replicates real life experience. Lastly, the distribution of patients in each treatment group was uneven which limits comparison. The largest group was treated with methotrexate based combined chemotherapy and had a prolonged overall survival.

When compared to similar prior studies, the strengths of our study include: 1) a larger, multicenter sample size with the inclusion of PLWH; 2) the use of co-registered T1 post-contrast images to ensure of whole tumor analysis instead of a singular or multiple regions of interest; 3) normalizing ADC values to normal contralateral white matter; 4) stratifying patients based on immune status, which to our knowledge, has not been extensively evaluated; and 5) inclusion of imaging and clinical characteristics which demonstrated an impact on outcomes.

The role of MR in PCNSL historically has been the detection and qualitative evaluation of response to treatment. DWI and derived ADC maps have been a well-established tool in neuroimaging, but the use of ADC histogram profiling has not been widely accepted in daily practice. Our data expands the role of conventional MR imaging by utilizing quantitative ADC histogram analysis to predict clinical outcomes and tumor expression of Ki-67, a biomarker for tumor proliferative activity, in immunocompetent PCNSL patients. The role of using ADC as an imaging biomarker in PLWH may be limited. Quantitative ADC histogram analysis should be strongly considered as part of the imaging protocol in the evaluation of immunocompetent patients with PCNSL.

## MATERIALS AND METHODS

### Patient selection

This is a retrospective IRB-approved, HIPAA-compliant study of patients diagnosed with PCNSL at The University of Texas MD Anderson Cancer Center (MDACC) between March 2000 and July 2016 and at Ben Taub Hospital (BTH) between January 2012 and December 2016. Patients that met the following inclusion criteria were included: 1) age > 18 years; 2) pathologically proven PCNSL that presented during the study period; 3) pretreatment standard brain MRI at 1.5T or 3.0T; 4) no evidence of systemic lymphoma by whole-body computed tomography or positron emission tomography scan, and bone marrow biopsy. We reviewed the electronic health records (EHR) and collected relevant socio-demographic characteristics, ECOG performance status, HIV status, pathology results, expression of Ki-67, MR imaging characteristics, treatment and clinical outcomes. The patient population selected for this study is identical to the population used in our prior study evaluating tumor histogram analysis in PCNSL [28].

### MR imaging parameters

Images were acquired using the clinical diagnostic parameters using either a 1.5 or 3.0 Tesla scanners, General Electric (Milwaukee, Wisconsin) or Siemens (Erlangen, Germany); a minority of the images were from outside facilities. DWI were obtained with the following parameters using spin echo, echo planar sequences: 1) for BTH: minimum echo time (TE), time (TR) = 8000 ms, no flip angle, slice thickness = 5 mm (27 slices), and field of view (FOV) = 36 cm with diffusion-sensitizing gradients applied with b factors of 0 and 1000 s/mm^2^ 2) for MDACC: TE = 8.9 ms, TR = 6600 ms, slice thickness = 5 mm, and FOV = 36 cm with diffusion-sensitizing gradients applied with b factors of 0, 500, and 1000 s/mm^2^. ADC maps were automatically generated on the operating console with commercially available software. Axial T1-weighted post contrast sequences were obtained with the following parameters: 1) for BTH: TE = 11ms, TR 750 ms, slice thickness = 5 mm, FOV = 24 cm and 354 × 192 acquisition matrix; 2) for MDA: 12ms TE, 700 TR, 5 mm slice thickness, FOV = 24 cm and 352 × 224 acquisition matrix.

### Analysis of MRI imaging

**Figure 3:**
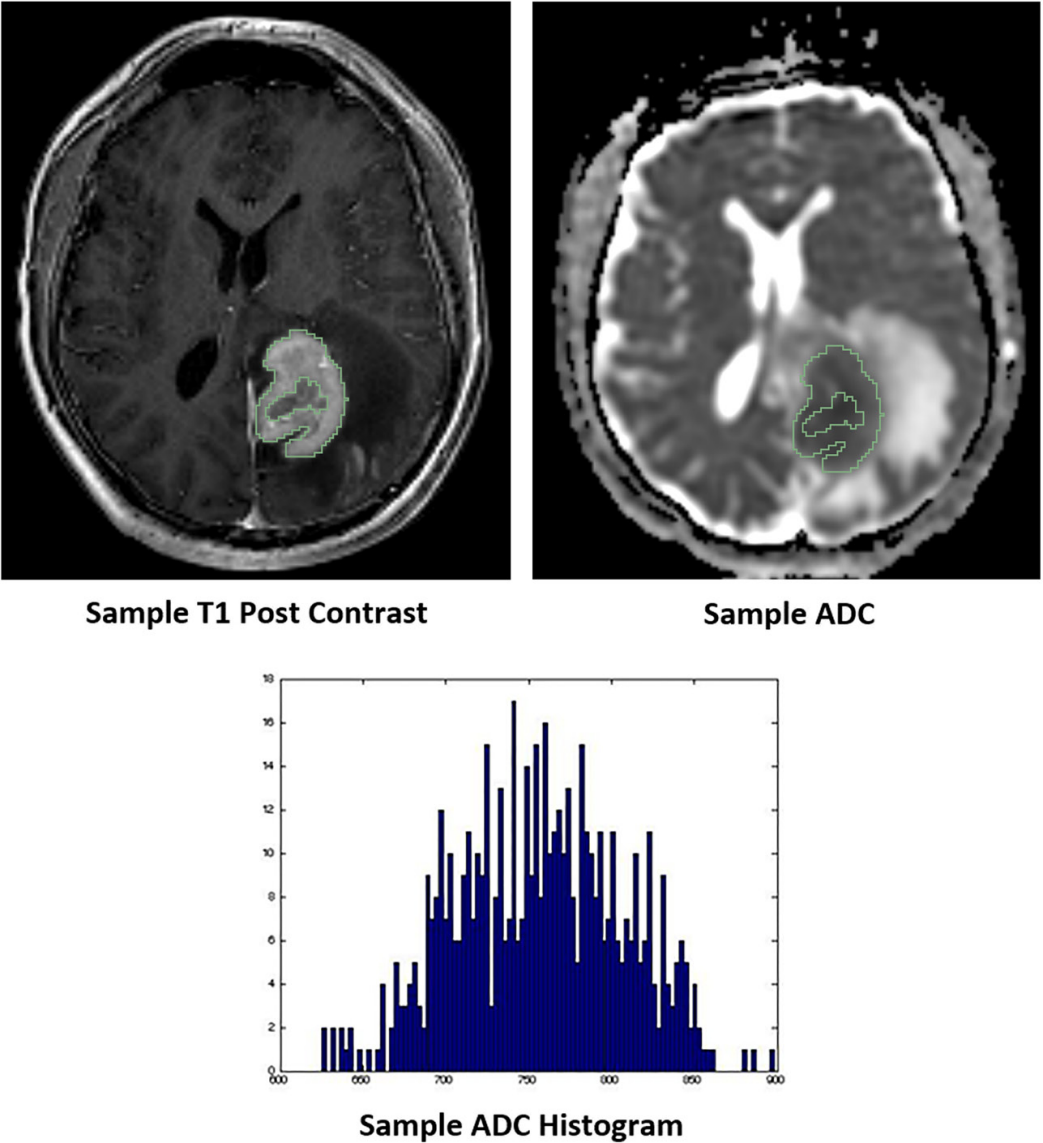
Representative images of whole tumor volume segmentation of the co-registered T1 post-contrast sequence and apparent diffusion coefficient (ADC) map, yielding the corresponding ADC histogram distribution utilized for data analysis.

Images were exported from the PACS in DICOM format, converted to a Neuroimaging Informatics Technology Initiative (NIfTI) file and then were uploaded to 3D slicer (version 4.7, http://www.slicer.org). This is an open source software platform for medical imaging informatics [40]. 3D slicer was used for image analysis and segmentation. Using 3D slicer, ADC maps and the T1 post-gadolinium contrast sequences were co-registered. Volume of interest (VOI) consisting of areas of gadolinium-enhancement were outlined and automatically propagated onto the corresponding ADC map (Figure 3). Only one lesion was selected for analysis in each patient. For patients with multiple lesions, the largest lesion was used for analysis. The VOI was segmented to exclude necrosis and hemorrhage in order to optimally obtain enhancing solid tumor. Necrosis was defined as an area of non-enhancement within the VOI. Hemorrhage was diagnosed by imaging features using pre-contrast T1WI and gradient echo sequences. To ensure optimum segmentation, 2 board certified neuroradiologists in consensus reviewed all tumor VOI prior to analysis (F. E. M. 15 years of experience, and R. R. C. 9 years of experience). A contralateral normal white matter VOI was obtained to calculate normalized ADC (nADC) ratios to correct for variations in imaging technologies and white matter disease within the dataset [14, 17, 21]. Distribution of the ADC values for the segmented tumors, normal white matter and normalized ADC values are provided as supplementary materials (Supplementary Figures 1–3).

nADC ratios were calculated as the ratio of the ADC values within the lesion to the ADC value of normal white matter. Subsequently, an in-house software built on Matlab was used to obtain the following histogram features: minimum, maximum, mean, standard deviation, skewness, kurtosis and the various ADC percentiles (1st, 5th, 15th, 25th, 75th, 95th, and 99th) of the ADC map VOIs. The volume of the VOI was obtained by multiplying the number of voxels within the VOI with the voxel size. Qualitative analysis was subsequently performed. This included the classification of tumor lesions with and without intra-tumoral hemorrhage, with ring versus solid enhancement pattern, and deep brain involvement, which included periventricular regions, basal ganglia, brainstem and/or cerebellum.

### Clinical outcomes

Two clinical outcomes, PFS and OS, were analyzed. We calculated the OS from the time of diagnosis to death of any cause. We calculated the PFS from the time of treatment to progression, relapse, or death from any cause, whichever occurred first. Complete remission was defined according to the International Primary Central Nervous System Lymphoma Collaboration Group Response Criteria [37, 41]. OS and PFS were correlated for each treatment group, including: 1) Supportive therapy, 2) Whole-brain radiation (WBRT), 3) methotrexate (MTX) monotherapy, 4) MTX-based combination chemotherapy, 5) WBRT plus MTX-based chemotherapy and 6) autologous stem cell transplantation (SCT). Of the 90 patients that met selection criteria of this study, one patient was excluded in analysis of these two clinical outcomes due to lack of available ECOG score.

### Statistical analysis

Analysis of demographic data and imaging characteristics by HIV status were performed using Pearson’s chi-squared test and Fisher’s exact test when any group contained four or less individuals. Median survival time was generated using Kaplan-Meier methods and *p*-values were calculated using Cox Proportional Hazards models. Normalized ratios were generated by dividing the tumor value against the corresponding white matter value. Correlations between nADC parameters and Ki-67 were generated using Pearson’s correlation coefficient. Correlations between non-normalized ADC parameters and Ki-67 were also performed for completeness (Supplementary Table 2). False discovery rate adjustment of these correlations were performed for 9 tests in 3 groups using Benjamini & Hochberg method (Supplementary Table 3). Associations between nADC parameters and OS and PFS were performed using Cox Proportional Hazards models adjusted for age at diagnosis, ECOG score, HIV status, and treatment. One patient was excluded from the Cox Proportional Hazards models due to a lack of available ECOG score. For completeness, associations between non-normalized ADC parameters and OS and PFS were similarly performed and included as supplementary material (Supplementary Tables 4 and 5). Hazard ratios (HR) and 95% confidence intervals (95%CI) were generated using values that were divided at the median. Statistical analyses were performed using R 3.5.0 [42]. Statistical significance was set to a *p* value < 0.05.

## SUPPLEMENTARY MATERIALS





## Abbreviations

PCNSL: Primary central nervous system lymphoma
DWI: Diffusion weighted imaging
ADC: Apparent diffusion coefficient
VOI: Volume of interest
nADC: Normalized ADC
PLWH: Patients living with HIV
MDACC: MD Anderson Cancer Center
BTH: Ben Taub Hospital
SCT: Stem cell transplant
ECOG: Eastern Cooperative Oncology performance status
WM: white matter
