# Supplementary material for: Diffusion-weighted MR imaging histogram analysis in HIV positive and negative patients with primary central nervous system lymphoma as a predictor of outcome and tumor proliferation
Source: Oncotarget. 2020 Nov 10;11(45):4093–103. doi: 10.18632/oncotarget.27800 (PMC7665236; doi:10.18632/oncotarget.27800)
Supplement: Supplementary file 1 [file oncotarget-11-4093-s001.pdf]

Diffusion-weighted MR imaging histogram analysis in HIV positive and negative patients with primary central nervous system lymphoma as a predictor of outcome and tumor proliferation

SUPPLEMENTARY MATERIALS

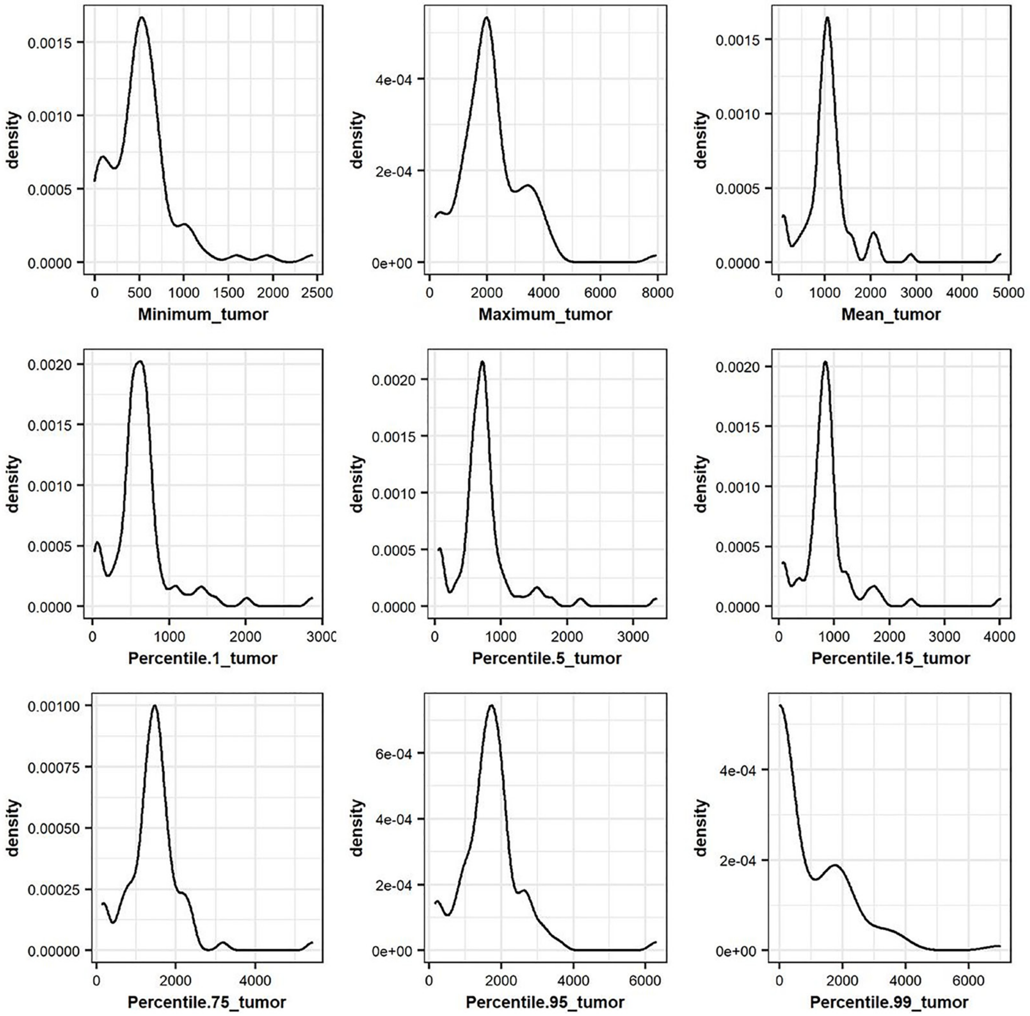

Supplementary Figure 1: Distribution of segmented tumor ADC values in all patients.

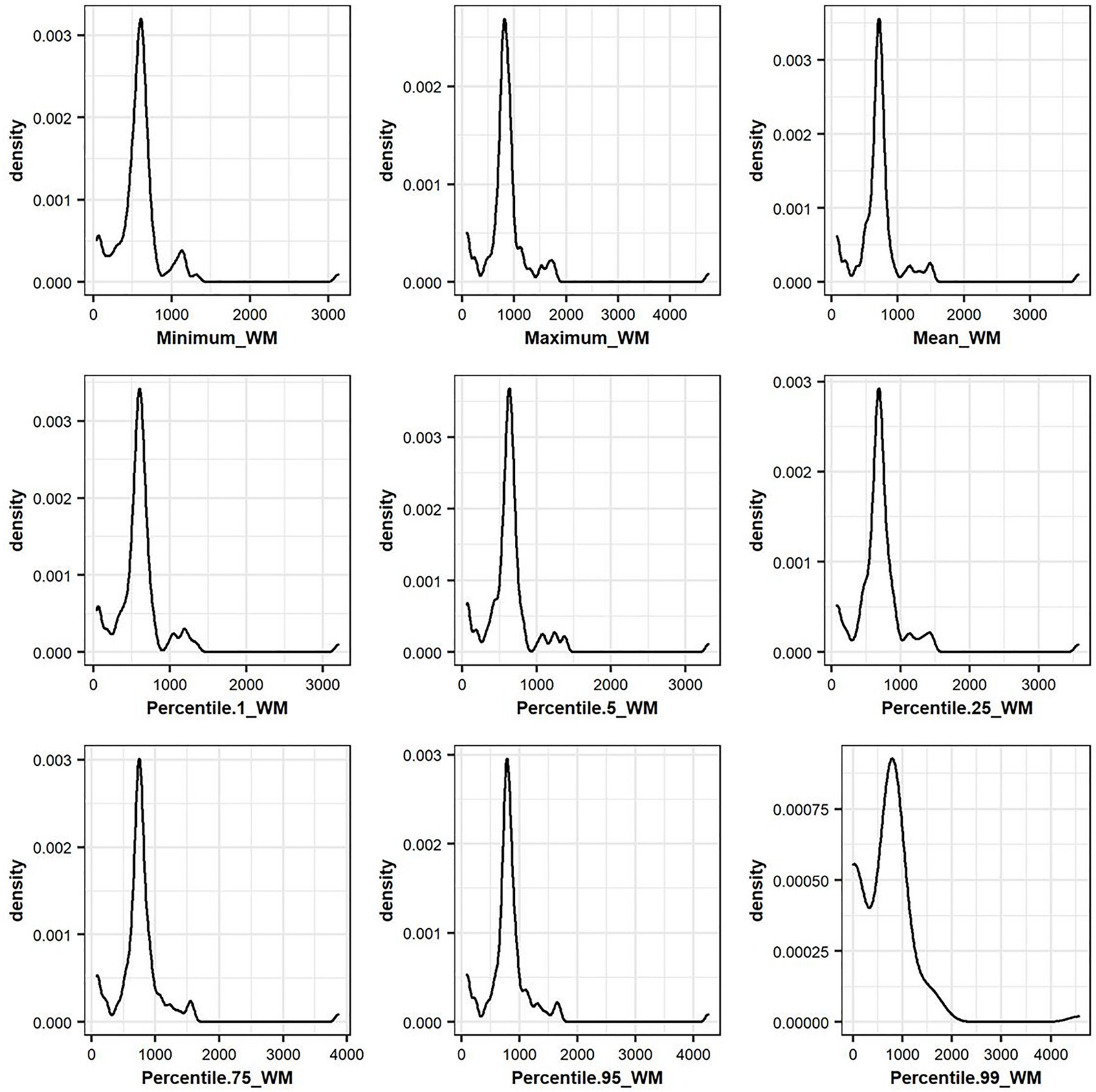

Supplementary Figure 2: Distribution of normal white matter ADC values in all patients.

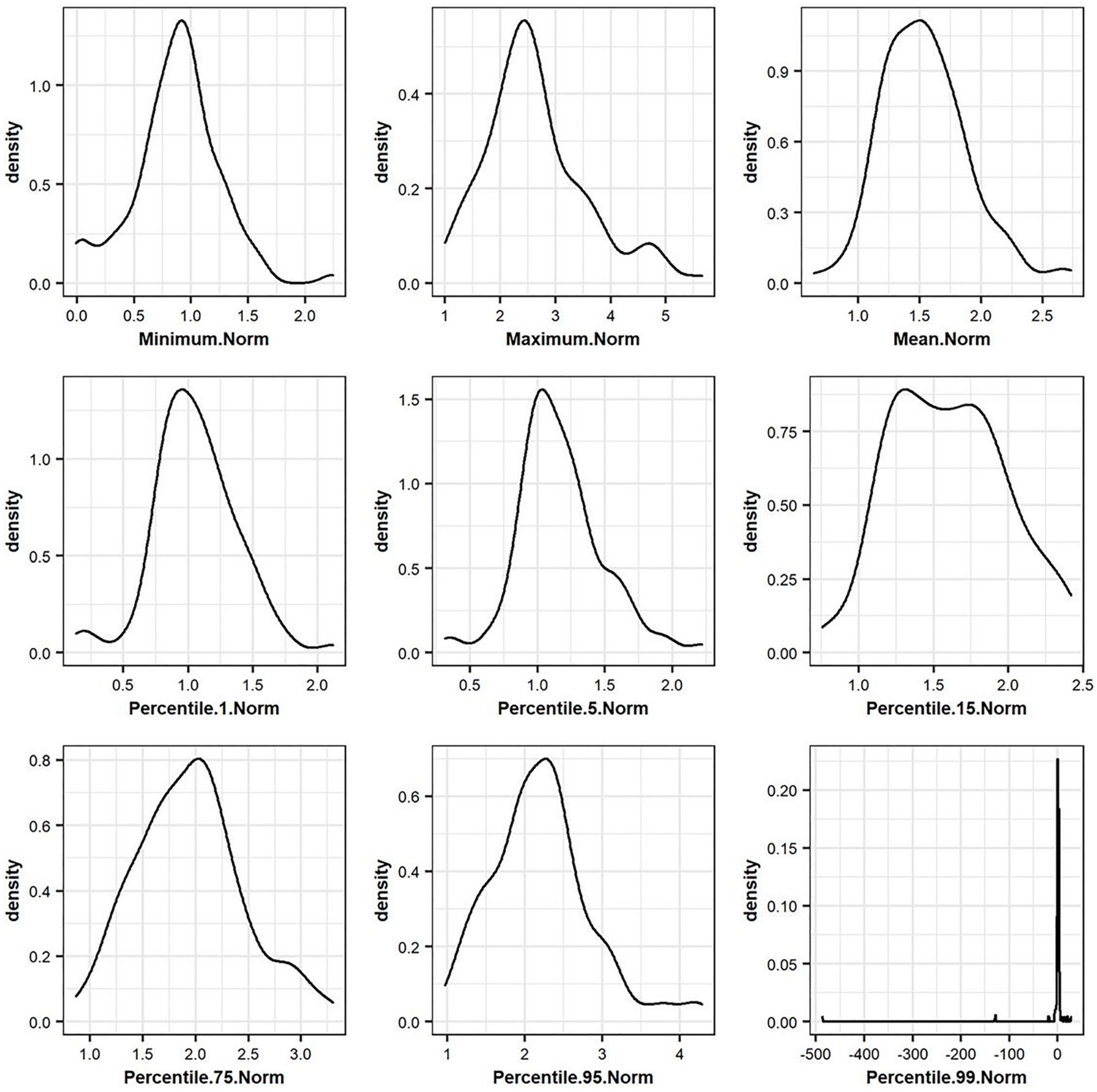

Supplementary Figure 3: Distribution of normalized ADC values in all patients.

Supplementary Table 1: Imaging characteristics and predictors of overall survival and progression free survival among all patients with PCNSL. See Supplementary Table 1

Supplementary Table 2: Correlation with Ki-67 in all patients and patients with and without HIV with non-normalized ADC parameters

Parameter	All patients (n = 22)			HIV positive (n = 3)			HIV negative (n = 19)		
	r	p-value	FDR-adjusted p value[1]	r	p-value	FDR-adjusted p value[1]	r	p-value	FDR-adjusted p value[1]
ADC_{Min}	0.168	0.4559	0.9329	0.188	0.4400	0.9329	–0.174	0.8884	0.9329
ADC_{Max}	0.078	0.7286	0.9329	0.105	0.6701	0.9329	–0.144	0.9082	0.9329
ADC_{Mean}	0.112	0.6190	0.9329	0.137	0.5750	0.9329	–0.206	0.8682	0.9329
ADC_1	0.165	0.4633	0.9329	0.183	0.4528	0.9329	–0.105	0.9329	0.9329
ADC_5	0.147	0.5141	0.9329	0.169	0.4886	0.9329	–0.150	0.9040	0.9329
ADC_{15}	0.109	0.6291	0.9329	0.134	0.5851	0.9329	–0.233	0.8505	0.9329
ADC_{75}	0.079	0.7268	0.9329	0.112	0.6476	0.9329	–0.325	0.7890	0.9329
ADC_{95}	0.095	0.6744	0.9329	0.126	0.6083	0.9329	–0.239	0.8466	0.9329
ADC_{99}	0.199	0.3752	0.9329	0.200	0.4114	0.9329	0.189	0.8790	0.9329

[1]Adjusted for 9 tests in 3 groups using Benjamini & Hochberg method. Results considered significant (*) when $p < 0.05$.

Supplementary Table 3: Correlation with Ki-67 in all patients and patients with and without HIV with inclusion of false discovery rate adjustment

Parameter	All patients (n = 22)			HIV positive (n = 3)			HIV negative (n = 19)		
	r	p-value	FDR-adjusted p value[1]	r	p-value	FDR-adjusted p value[1]	r	p-value	FDR-adjusted p value[1]
Skewness	−0.130	0.5641	0.6132	−0.666	0.5361	0.6100	−0.102	0.6785	0.6785
Kurtosis	−0.226	0.3125	0.5287	−0.901	0.2859	0.5272	−0.167	0.4957	0.6037
$nADC_{Min}$	0.244	0.2737	0.5272	0.973	0.1486	0.5272	0.193	0.4284	0.6037
$nADC_{Max}$	−0.259	0.2442	0.5272	0.725	0.4834	0.6037	−0.323	0.1775	0.5272
$nADC_{Mean}$	−0.237	0.2876	0.5272	0.955	0.1928	0.5272	−0.494	0.0317*	0.4297
$nADC_1$	0.290	0.1907	0.5272	0.702	0.5050	0.6037	0.241	0.3204	0.5287
$nADC_5$	0.199	0.3744	0.5681	0.617	0.5766	0.6132	0.130	0.5946	0.6132
$nADC_{15}$	−0.394	0.0695	0.5272	0.828	0.3787	0.5681	−0.580	0.0092*	0.3044
$nADC_{75}$	−0.349	0.1118	0.5272	0.942	0.2185	0.5272	−0.477	0.0391*	0.4297
$nADC_{95}$	−0.267	0.2297	0.5272	0.991	0.0849	0.5272	−0.358	0.1325	0.5272
$nADC_{99}$	−0.148	0.5122	0.6037	0.921	0.2551	0.5272	−0.175	0.4743	0.6037

[1]Adjusted for 11 tests in 3 groups using Benjamini & Hochberg method. Results considered significant (*) when $p < 0.05$.

Supplementary Table 4: Non-normalized ADC hazard ratios for overall survival in all patients and patients with and without HIV

Feature	All patients (n = 89)[1]		HIV+ (N = 23)[2]		HIV- (n = 66)[2]	
	p-value	HR (95% CI)	*p*-value	HR (95% CI)	*p*-value	HR (95% CI)
ADC_{Min}	0.8508	0.945 (0.522–1.71)	0.2323	2.083 (0.625–6.941)	0.2460	0.645 (0.308–1.353)
ADC_{Max}	0.6129	1.17 (0.637–2.146)	0.9306	0.955 (0.341–2.672)	0.1433	1.807 (0.818–3.992)
ADC_{Mean}	0.1461	0.617 (0.322–1.183)	0.8042	0.837 (0.205–3.422)	0.1501	0.559 (0.254–1.234)
ADC_1	0.9162	0.969 (0.537–1.748)	0.4652	1.554 (0.476–5.074)	0.5549	0.8 (0.381–1.678)
ADC_5	0.6634	1.137 (0.638–2.028)	0.5363	1.41 (0.475–4.184)	0.9134	1.041 (0.508–2.133)
ADC_{15}	0.7213	1.113 (0.617–2.009)	0.4793	1.516 (0.479–4.802)	0.8996	0.954 (0.459–1.984)
ADC_{75}	0.9438	0.978 (0.534–1.793)	0.6284	1.338 (0.411–4.358)	0.9795	1.01 (0.476–2.145)
ADC_{95}	0.7517	0.909 (0.501–1.646)	0.4645	1.498 (0.507–4.42)	0.7347	0.876 (0.409–1.88)
ADC_{99}	0.0230*	0.464 (0.24–0.899)	0.0734	0.263 (0.061–1.135)	0.3033	0.663 (0.303–1.45)

[1]Adjusted for age, ECOG, HIV status, treatment. [2]Adjusted for age, ECOG, treatment. Results considered significant (*) when $p < 0.05$.

Supplementary Table 5: Non-normalized ADC hazard ratios for progression free survival in all patients and patients with and without HIV

Feature	All patients (n = 89)[1]		HIV+ (N = 23)[2]		HIV- (n = 66)[2]	
	p-value	HR (95% CI)	*p*-value	HR (95% CI)	*p*-value	HR (95% CI)
ADC_{Min}	0.7686	0.917 (0.516–1.63)	0.3150	1.892 (0.545–6.563)	0.1657	0.617 (0.312–1.221)
ADC_{Max}	0.3630	1.307 (0.734–2.327)	0.5914	1.336 (0.464–3.853)	0.3749	1.365 (0.686–2.717)
ADC_{Mean}	0.0919	0.605 (0.337–1.085)	0.9366	0.945 (0.237–3.772)	0.0308*	0.445 (0.214–0.928)
ADC_1	0.8255	1.065 (0.607–1.871)	0.5949	1.395 (0.409–4.758)	0.6678	0.865 (0.447–1.675)
ADC_5	0.5629	1.177 (0.677–2.045)	0.6174	1.328 (0.437–4.038)	0.9369	1.027 (0.533–1.977)
ADC_{15}	0.4556	1.241 (0.703–2.191)	0.6297	1.329 (0.418–4.22)	0.7121	1.134 (0.581–2.216)
ADC_{75}	0.3336	1.337 (0.742–2.409)	0.3497	1.869 (0.504–6.935)	0.9635	0.983 (0.482–2.009)
ADC_{95}	0.3962	1.284 (0.721–2.287)	0.1723	2.233 (0.705–7.074)	0.7875	0.908 (0.45–1.832)
ADC_{99}	0.0172*	0.477 (0.259–0.877)	0.1003	0.311 (0.077–1.252)	0.1129	0.575 (0.29–1.14)

[1]Adjusted for age, ECOG, HIV status, treatment. [2]Adjusted for age, ECOG, treatment. Results considered significant (*) when $p < 0.05$.
