# Supplementary material for: Diffusion-weighted MR imaging histogram analysis in HIV positive and negative patients with primary central nervous system lymphoma as a predictor of outcome and tumor proliferation
Source: Oncotarget. 2020 Nov 10;11(45):4093–103. doi: 10.18632/oncotarget.27800 (PMC7665236; doi:10.18632/oncotarget.27800)
Supplement: Supplementary file 2 [file oncotarget-11-4093-s002.docx]

**Supplementary Table 1: Imaging characteristics and predictors of overall survival and progression free survival among all patients with PCNSL**

|  |  |  |  |  | **Overall survival (in months)** | | | | **Progression-free survival (in months)** | | |
| --- | --- | --- | --- | --- | --- | --- | --- | --- | --- | --- | --- |
|  | **All patients** (n=90) | **HIV positive** (n=23, 25.6%) | **HIV negative** (n=67, 74.4%) | **p-value** | **Deaths** | **Median in months (95% CI)** | **p-value** | **Relapses** | | **Median in months (95% CI)** | **p-value** |
| **Total** | 90 | 23 (25.6%) | 67 (74.4%) |  | 52 | 41 (23-52) |  | 55 | | 11 (6-24) |  |
| **Study site** |  |  |  | 2.39 × 10^−12^ |  |  |  |  | |  |  |
| BTH | 32 (35.6%) | 22 (95.7%) | 10 (14.9%) |  | 21 | 15 (5-43) | Ref | 23 | | 6 (2-15) | Ref |
| MDACC | 58 (64.4%) | 1 (4.3%) | 57 (85.1%) |  | 31 | 47 (37-101) | 0.0175 | 32 | | 15 (9-**) | 0.041 |
| **Gender** |  |  |  | 0.1485 |  |  |  |  | |  |  |
| Male | 49 (54.4%) | 16 (69.6%) | 33 (49.3%) |  | 32 | 35 (15-47) | Ref | 33 | | 7 (3-33) | Ref |
| Female | 41 (45.6%) | 7 (30.4%) | 34 (50.7%) |  | 20 | 45 (24-**) | 0.0821 | 22 | | 13 (8-**) | 0.137 |
| **Age** |  |  |  | 7.22 x10^−8^ |  |  |  |  | |  |  |
| <60 | 50 (55.6%) | 23 (100.0%) | 27 (40.3%) |  | 30 | 41 (24-**) | Ref | 30 | | 12 (6-**) | Ref |
| ≥60 | 40 (44.4%) | 0 (0.0%) | 40 (59.7%) |  | 22 | 41 (16-**) | 0.944 | 25 | | 11 (6-**) | 0.982 |
| **Race/Ethnicity** |  |  |  | 5.78 x10^−8^ |  |  |  |  | |  |  |
| Non-Hispanic/White | 44 (48.9%) | 2 (8.7%) | 42 (62.7%) |  | 29 | 41 (21-53) | Ref | 28 | | 11 (6-**) | Ref |
| African-American | 19 (21.1%) | 14 (60.9%) | 5 (7.5%) |  | 14 | 6 (5-**) | 0.219 | 12 | | 6 (3-**) | 0.698 |
| Hispanics | 24 (26.7%) | 7 (30.4%) | 17 (25.4%) |  | 8 | ** | 0.134 | 13 | | 17 (10-**) | 0.445 |
| Asian | 3 (3.3%) | 0 (0.0%) | 3 (4.5%) |  | 1 | ** | 0.679 | 2 | | 6 (5-**) | 0.944 |
| **HIV status** |  |  |  |  |  |  |  |  | |  |  |
| Positive | 23 (25.6%) | – | – |  | 16 | 6 (2-**) | Ref | 16 | | 5 (2-**) | Ref |
| Negative | 67 (74.4%) | – | – |  | 36 | 43 (37-100) | 0.0032 | 39 | | 13 (9-**) | 0.0684 |
| **ECOG** |  |  |  | 2.78 × 10^−5^ |  |  |  |  | |  |  |
| 0-1 | 49 (54.4%) | 4 (17.4%) | 45 (67.2%) |  | 22 | 47 (43-**) | Ref | 25 | | 24 (11-**) | Ref |
| 2-4 | 40 (44.4%) | 19 (82.6%) | 21 (31.3%) |  | 29 | 8 (5-37) | 3.46 × 10^−5^ | 30 | | 5 (2-13) | 0.00314 |
| Not available | 1 (1.1%) | 0 (0.0%) | 1 (1.5%) |  |  |  |  |  | |  |  |
| **Number of lesions** |  |  |  | 0.0103 |  |  |  |  | |  |  |
| Single | 50 (55.6%) | 7 (30.4%) | 43 (64.2%) |  | 30 | 43 (24-53) | Ref | 33 | | 8 (14-5-**) | Ref |
| Multiple | 40 (44.4%) | 16 (69.6%) | 24 (35.8%) |  | 22 | 37 (9-**) | 0.729 | 22 | | 14 (6-**) | 0.434 |
| **Location** |  |  |  | 0.7104 |  |  |  |  | |  |  |
| Deep brain | 42 (46.7%) | 12 (52.2%) | 30 (44.8%) |  | 27 | 37 (11-100) | Ref | 28 | | 11 (6-24) | Ref |
| Not deep brain | 48 (53.3%) | 11 (47.8%) | 37 (55.2%) |  | 25 | 41 (24-**) | 0.72 | 27 | | 12 (6-**) | 0.599 |
| **Hemorrhage** |  |  |  | 0.1383 |  |  | 0.0106 |  | |  | 0.0548 |
| Yes | 40 (44.4%) | 15 (65.2%) | 25 (37.3%) |  | 30 | 15 (6-43) |  | 30 | | 5.5 (4-21) |  |
| No | 40 (44.4%) | 8 (34.8%) | 32 (47.8%) |  | 21 | 45 (37-**) |  | 22 | | 14 (9-**) |  |
| Not available | 10 (11.1%) | 0 (0.0%) | 10 (14.9%) |  | – | – |  | – | | – |  |
| **Enhancement** |  |  |  | 8.57 × 10^−9^ |  |  |  |  | |  |  |
| Ring | 15 (16.7%) | 13 (56.5%) | 2 (3.0%) |  | 11 | 5 (2-**) | Ref | 10 | | 5 (2-**) | Ref |
| Solid | 74 (82.2%) | 9 (39.1%) | 65 (97.0%) |  | 41 | 43 (27-53) | 0.0814 | 45 | | 11 (7-**) | 0.281 |
| None | 1 (1.1%) | 1 (4.3%) | 0 (0.0%) |  | – | – |  | 55 | | 11 (6-24) |  |
|  |  |  |  |  |  |  |  |  | |  |  |
| **Initial Treatment** |  |  |  | 2.68 × 10^−10^ |  |  |  |  | |  |  |
| Supportive | 5 (5.6%) | 3 (13.0%) | 2 (3.0%) |  | 5 | 1 (1-**) | Ref | 5 | | 1 (1-**) | Ref |
| WBRT | 17 (18.9%) | 15 (65.2%) | 2 (3.0%) |  | 11 | 24 (3-**) | 3.09 × 10^−5^ | 10 | | 6 (3-**) | 5.85 × 10^−5^ |
| MTX monotherapy | 5 (5.6%) | 0 (0.0%) | 5 (7.5%) |  | 5 | 8 (3-**( | 0.00636 | 5 | | 3 (2-**) | 0.0319 |
| MTX-based combination chemo | 52 (57.8%) | 5 (21.7%) | 47 (70.1%) |  | 27 | 43 (35-**) | 2.88 × 10^−8^ | 32 | | 13 (9-**) | 1.89 × 10^−6^ |
| WBRT and MTX based chemo | 11 (12.2%) | 0 (0.0%) | 11 (16.4%) |  | 4 | 100 (47-**) | 8.29 × 10^−8^ | 3 | | ** | 3.68 × 10^−6^ |
| **SCT** |  |  |  | 0.0495 |  |  |  |  | |  |  |
| Auto-HSCT | 11 (12.2%) | 0 (0.0%) | 11 (16.4%) |  | 3 | 76 (47-**) | Ref | 3 | | ** | Ref |
| No SCT | 79 (87.8%) | 23 (100.0%) | 56 (83.6%) |  | 49 | 37 (11-45) | 0.0401 | 52 | | 8 (5-**) | 0.0195 |

Abbreviations: BTH, Ben Taub Hospital; MDACC, MD Anderson Cancer Center; ECOG, Eastern Cooperative Oncology Group performance status; WBRT, whole brain radiation therapy; SCT, stem cell transplant.
